# Building a Children’s Health Service and System Research Strategy: development and integration in an Australian paediatric healthcare setting

**DOI:** 10.1186/s12913-020-05267-6

**Published:** 2020-06-29

**Authors:** Robyn Littlewood, Oliver J. Canfell, Frank Tracey

**Affiliations:** 1 Health and Wellbeing Queensland, Queensland Government, The State of Queensland, 139 Coronation Drive, Milton, QLD 4064 Australia; 2 School of Human Movement and Nutrition Sciences, Faculty of Health and Behavioural Sciences, The University of Queensland, St Lucia, QLD 4067 Australia; 3 Children’s Health Queensland Hospital and Health Service, Department of Health, Queensland Government, The State of Queensland, South Brisbane, QLD 4101 Australia; 4 Faculty of Medicine, The University of Queensland, St Lucia, QLD 4067 Australia

**Keywords:** Health services research, Health system, Health policy, Research strategy, Child

## Abstract

**Background:**

Health services and systems research (HSSR) strategies dedicated to paediatric health care and service delivery are limited. Strategies are available but are outdated and yet to be optimised for use in a paediatric health system. We aim to describe the development and integration of a Children’s Health Service and System Research Strategy (CHSSR-S) in Children’s Health Queensland (CHQ), a large specialist quaternary hospital and health service caring for children and young people in Queensland and northern New South Wales, Australia.

**Methods:**

The CHSSR-S was developed using an inductive, bottom-up, participatory systems approach across three phases: (1) Identifying local HSSR capacity; (2) Development; (3) Integration. A HSSR “Champion” was appointed to lead all phases. Clinical, research and system-based stakeholders (*n* = 14) were individually identified, contacted and participated in dedicated meetings and a workshop to iteratively design the CHSSR-S. A health system-wide CHSSR-S governance committee was established to drive phase three. Health system integration was achieved by multicomponent, action-based strategies.

**Results:**

The final CHSSR-S comprised ten Research Priorities and three Research Enablers, and was successfully integrated within CHQ via a range of platforms. Research Priorities included: (1) Population Health; (2) Adolescent and Young Adult (AYA) Cancer; (3) Indigenous Health; (4); Mental Health; (5) Nutrition and Obesity; (6) Rare Neurodevelopmental Disorders; (7) Sepsis; (8) Screening, surveillance and monitoring; (9) Innovation; and (10) Electronic Medical Record (EMR). Research Priorities were supported by three Research Enablers: (1) Data; (2); Evaluation and Health Economics; and (3) Policy.

**Conclusions:**

The CHSSR-S is the first known paediatric HSSR strategy developed and integrated within a large dedicated paediatric health system. The CHSSR-S may be used to guide global paediatric healthcare systems to prioritise HSSR in their local setting to optimise health service delivery and patient outcomes.

## Background

Health services research (HSR) has been described as the “innovation engine” of a health care system. It is a “*multi-disciplinary research activity with an implicit objective of improving the health services patients receive”* [1], with outcomes typically generated at a population, rather than individual, level. HSR is a contemporary and evolving approach that targets various elements of the healthcare system, often in congruence and underpinned by a multidisciplinary approach, including: quality of care (incorporating effectiveness, timeliness, and appropriateness), patient safety; accessibility of care; equity in healthcare delivery; health economics (i.e. cost-effectiveness, cost-benefits); and health service implementation and evaluation [2].

Previously in Australia, a significant proportion of health and medical research has focused on products, drugs and interventional acute care [3]. Recently, HSR has emerged as a nationwide priority area. In 2016, the Australian Government Department of Health’s Medical Research Future Fund (MRFF), a $20 billion investment vehicle for health and medical research, defined one of six key strategic directions to “strengthen [our] health services and systems research to make healthcare more efficient and affordable” [3]. Briefly, the public health care system in Australia is a national, universal scheme administered at the State level within a national framework [4]. A private health care system exists concurrently, comprising approximately 40% of all hospital admissions [4, 5].

The competing nature of public and private systems in Australia encourages inequity in cost and accessibility; people representing low socioeconomic areas exhibit the highest separation rates from public hospitals [5]. This is a significant barrier to high quality health service delivery in Australia. Paediatric health expenditure is viewed as particularly financially inefficient; children with medical complexity represent only 9.9% of total public hospital admissions, yet incur almost one-third (32.1%) of total public hospital costs [6]. Accessibility to services is an additional barrier to maximising care delivery; approximately 10% of Australian parents/guardians report difficulty in accessing health services [7]. Children with Aboriginal and Torres Strait Islander descent are also significantly less likely to access health services at critical developmental time points, particularly child health nurses and dentists in the early years [7]. Collectively, these issues highlight the growing requirement for value-based prevention, treatment and research solutions to healthcare in Australia [8, 9], a need that is inherently addressed in health services research principles [10].

Globally, policy-relevant HSR agendas that are integrated in a health service or system are rare, particularly those targeting child and youth. The Canadian Institute of Health Services and Policy Research (CIHSPR) is one example of a long-standing, outcomes-driven HSR agenda. The CIHSPR is led by a national health research body and has successfully mediated a significant increase in HSR-specific funding for Canada since 2000 [11]. However, the CIHSPR is an external, independent organisation to the Canadian health system and may be ill-positioned to create and sustain HSR practices across the system. Another example of a siloed HSR agenda is that developed by Fairbrother et al. [12] for children in the USA. Whilst robust in design, there was no application or evidence of engagement with a specific healthcare system.

Children’s Health Queensland (CHQ) is a statewide public hospital and health service providing community- and quaternary-level care as well as population-based services to children and young people (aged < 18 years) across the states of Queensland (and including some parts of Northern New South Wales), Australia. CHQ delivers an integrated network of services through the Queensland Children’s Hospital (QCH), Child and Youth Community Health Service (CYCHS) and Child and Youth Mental Health Service (CYHMS), as well as partnerships with other health services, non-government organisations and charity partners [13]. In 2018–19: QCH treated 17,000 inpatients and provided 290,977 occasions of outpatient care; CYCHS delivered 94,970 service contacts across 576 sites in Queensland; and CYMHS provided 8222 occasions of service [13]. In terms of research, CHQ partners with leading universities (The University of Queensland and Queensland University of Technology) and health organisations at the Centre for Children’s Health Research (South Brisbane, Australia). In 2015–16, the Queensland Government spent 2.6% (AUD$918 million) of recurrent spending on health and medical research [14].

CHQ identified that to support and prove impact and sustainability of their hospital and health service, an integrated HSR strategy that linked clinical and research systems was necessary. This strategy was seen as a first step to creating a HSR-driven environment that enabled the hospital and health service to understand true cost and liabilities, strengths, weaknesses and gaps to focus continued, iterative efforts to improve the system. There has been little known action to develop and integrate an Australian, healthcare system-wide paediatric HSR strategy, with a focus on improving efficiency, creating equity and tackling vulnerability in priority populations for a true life-course approach.

The objective of this article is to describe the development and integration of a paediatric HSR strategy within a dedicated community and quaternary paediatric health system. We propose the ‘system’ should also be recognised as priority. The health system should be the driver when considering HSR [15] and in designing and implementing a HSR strategy. A Health Service and System in its entirety, including strategy, planning, policy, procurement, finance, clinical service delivery, quality and safety should underpin the development of a collaborative Health Service and System Research (HSSR) strategy.

## Methods

### Setting

A hospital and health service (CHQ) dedicated to caring for children and young people across Queensland (including some parts of Northern New South Wales), Australia.

### Design

**Fig. 1:**
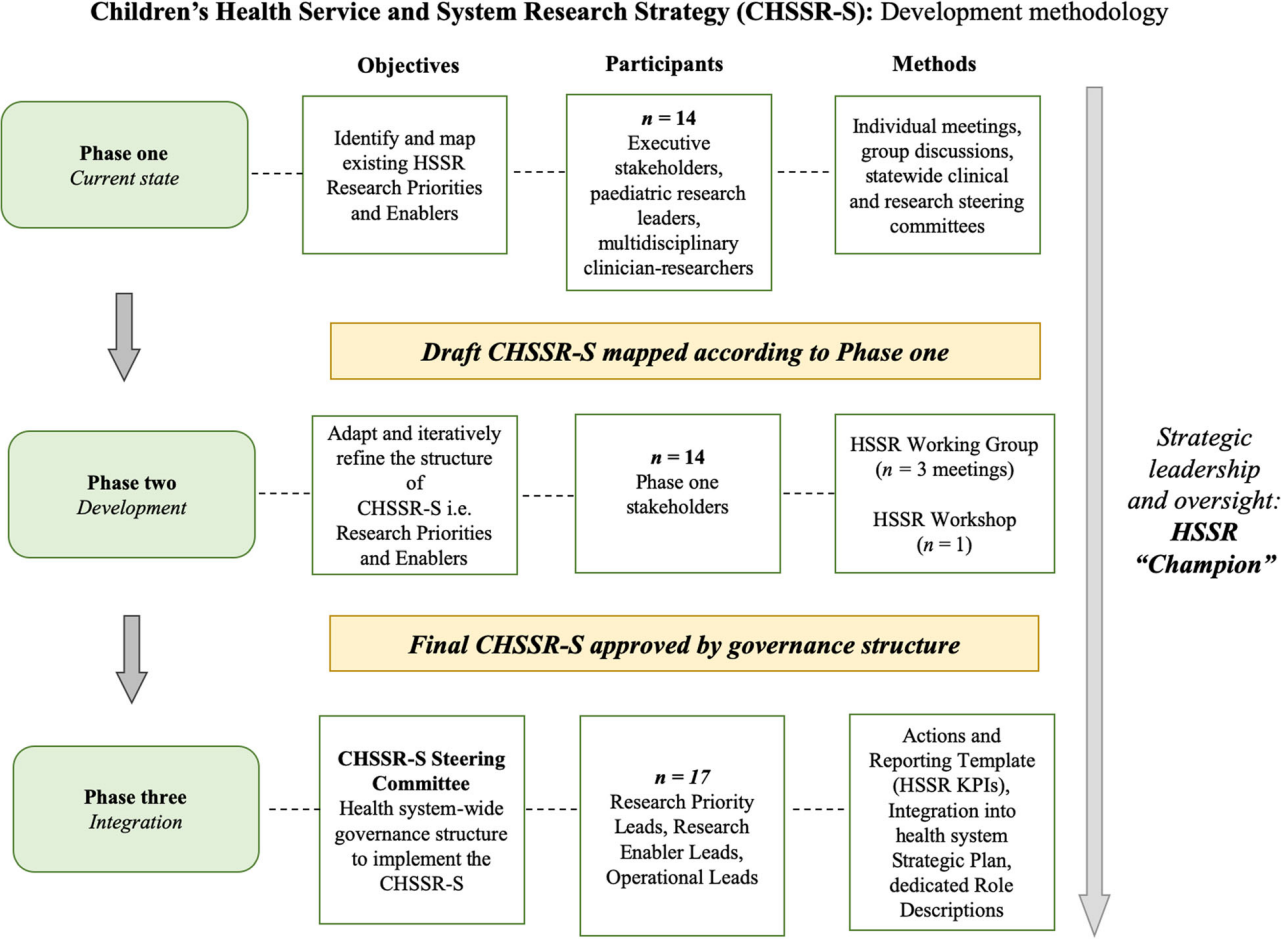
Children’s Health Service and System Research Strategy (CHSSR-S): Development methodology

Development of the Children’s Health Service and System Research Strategy (CHSSR-S) was informed by an inductive, bottom-up, participatory systems approach. This conceptual approach intended to place clinical, research and system leadership at the forefront of designing and implementing the CHSSR-S [16, 17]. This study was conducted across three key phases that were underpinned by our conceptual approach (see Fig. 1 for an overview of the purpose, participants and methodology of each phase):

1. *Identifying the current state of HSSR within the health system*
2. *Development of the CHSSR-S strategy*

The overall structure and content of the CHSSR-S was aligned with three interrelated dimensions of care relevant to HSSR [10]:

1. Decision-making – what is the best care to provide?
2. Implementation – how can we best provide this care?
3. Evaluation – what are the outcomes resulting from this care?
4. *Integration of the HSSR strategy within the health system*

These three key phases were underpinned by a stepwise consultation and iterative design process with key stakeholders, informed by recommendations for actioning stakeholder engagement in research [18]. Inputs were consolidated after the conclusion of each phase to inform a cumulative working draft of the CHSSR-S. The finalised CHSSR-S was agreed upon at the conclusion of phase two and was ready for health system integration in phase three. Implementation of each phase was driven by a dedicated HSSR “Champion” – a system leader, Research Director and allied health professional - for strategic oversight and to facilitate between-phase CHSSR-S iterative re-design.

#### Phase 1: identifying the current state of HSSR

The aim of phase one was to establish the “current state” of HSSR within the system by consolidating existing but fragmented local HSSR capacity. This involved identifying and mapping existing intra-organisational HSSR capacity.

The HSSR Champion identified key HSSR research leaders across a broad range of disciplines within the health system via one-on-one consultations with Executive and Research leaders, and Queensland-wide clinical and research steering committees. Initial contact with identified research leaders was then made via email correspondence or telephone calls by the HSSR Champion. Stakeholders in HSSR without research expertise were also identified. The role of the HSSR Champion was to generate understanding and interest in the HSSR strategic direction, build rapport with research leaders and stakeholders and explain roles and responsibilities in moving towards a health system-wide HSSR strategy – the CHSSR-S.

In phase 1, Research Priorities were mapped based on the identified research leaders, their existing research groups, research capacity, leadership and demonstration of impact. Through the initial consultation phase, it was determined that Research Priorities required dedicated support from “Research Enablers” to drive research success and translation. Research Enablers were themes of work designed to support robust data analysis and evaluation (Research Enablers: Data and Evaluation), influence research-informed health policy changes (Research Enabler: Policy) and ultimately drive the development of new research questions,. Research Enablers were seen as complementary and essential to the overarching CHSSR-S. See Table 1 for a description of phase 1 research leaders and stakeholders and their associated Research Priorities and Research Enablers.

**Table 1:** Phase 1 - Key HSSR research leaders and stakeholders in CHQ and their associated Research Priorities and Research Enablers

Leaders and stakeholders	*n* (%)	Research Priorities	Research Enablers
HSSR Champion	1 (7)	–	Policy
Executive Leader	1 (7)	Interprofessional education	–
Research Leader^a^	2 (14)	Mental Health AYA Cancer	–
Medical	4 (29)	Sepsis Population Health Integrated Care	Evaluation
Dietitian	2 (14)	Nutrition Obesity	–
Clinical Trials Coordinator	1 (7)	Rare Neurodevelopmental Disorders	–
Multicultural Health Coordinator	1 (7)	Indigenous Health	–
Data analyst	1 (7)	EMR	Data
Media & Communications Officer	1 (7)	–	–

*HSSR* Health Services and Systems Research, *AYA* Adolescent and Young Adult, *EMR* Electronic Medical Record

^a^Professor (Mental Health) and Research Fellow (AYA Cancer)

A foundational first draft of the CHSSR-S was then mapped according to identified Research Priorities and Research Enablers.

#### Phase 2: development of the CHSSR-S

The aim of phase two was to refine the foundational CHSSR-S draft from phase one into a progressive, definitive health system-based Strategy.

Phase one research leaders and stakeholders were recruited to participate in a HSSR Working Group, including additional stakeholders not identified in phase one (i.e. without a strong HSSR presence in CHQ), such as nursing and other clinical staff. Membership of this group was fluid and iteratively changed according to demands and results of the project i.e. adjustments to the CHSSR-S. Membership was conditional on an understanding of equity between stakeholders, Research Priorities and Research Enablers; no one area or stakeholder was deemed more important. As development of the CHSSR-S aligned with the general strategic direction for research in CHQ, members were expected to contribute an in-kind commitment to its inception and integration. The HSSR Working Group participated in 3 meetings, in addition to a dedicated workshop, over 5 months to develop, review and finalise the CHSSR-S. Throughout the development process, the following key “umbrella” questions (uniquely created for contextualisation to the present study) were consistently asked of HSSR Working Group members (see also Supplementary File):

- How can you change the system?
- What outcomes would you like to improve?
- What service gaps can you identify?
- How can you improve the value of your service?
- How can you influence statewide and national policy?
- How can you use innovation and digital health to evaluate services and inform delivery of care?
- Who can drive robust evaluation of your current services?

These questions were designed to refine the Research Priorities and Research Enablers identified in phase one, explore opportunities for consolidating Priorities or Enablers and identify new areas of need. Additionally, formal feedback about the iterative progression of the CHSSR-S was sought throughout phase two from well-established, mature consumer-led advisory committees led by CHQ, such as the Making Tracks Committee (dedicated to Aboriginal and Torres Strait Islander Health), the Family Advisory Council and the Family Centred Care Committee – all representing the interests of families using CHQ health services.

The CHSSR-S was subsequently refined via meeting minutes and group editing via email correspondence. Decisional power rested with a quorum of the HSSR Working Group. Any conflicted or tied opinions were resolved by decision of the HSSR Champion. After two dedicated meetings, a half-day HSSR Workshop was held to finalise the CHSSR-S, with representation from the entire HSSR Working Group or nominated proxies. A final draft of the CHSSR-S was then presented to the Working Group and Executive Leadership Team for approval and endorsement, respectively.

#### Phase 3: integration of the CHSSR-S within the health system

The aim of phase three was to practically implement the approved CHSSR-S and ensure its integration within the health system. Leaders of each Research Priority and Research Enabler were required to update (3-monthly) an Actions and Reporting Template, adapted from an existing framework to monitor HSR [19] key performance indicators (KPIs) to consider the acute “system”. This template was purposed for quantifying and reporting HSSR outputs and generating ownership to promote sustainability [20]. The HSSR Champion was also responsible for identifying and actioning organisation-level strategies to ensure successful health system integration. These were identified through Executive-level discussions and HSSR Working Group meetings and focused on integration within system-wide platforms to promote long-term sustainability.

The final action to drive health system integration of the CHSSR-S was transformation of the HSSR Working Group into a formal steering committee (CHSSR-S Steering Committee) with a dedicated Terms of Reference, Executive- and Board-level reporting requirements and standing membership of key research leaders and stakeholders.

## Results

### Children’s health service and system research strategy (CHSSR-S)

**Fig. 2:**
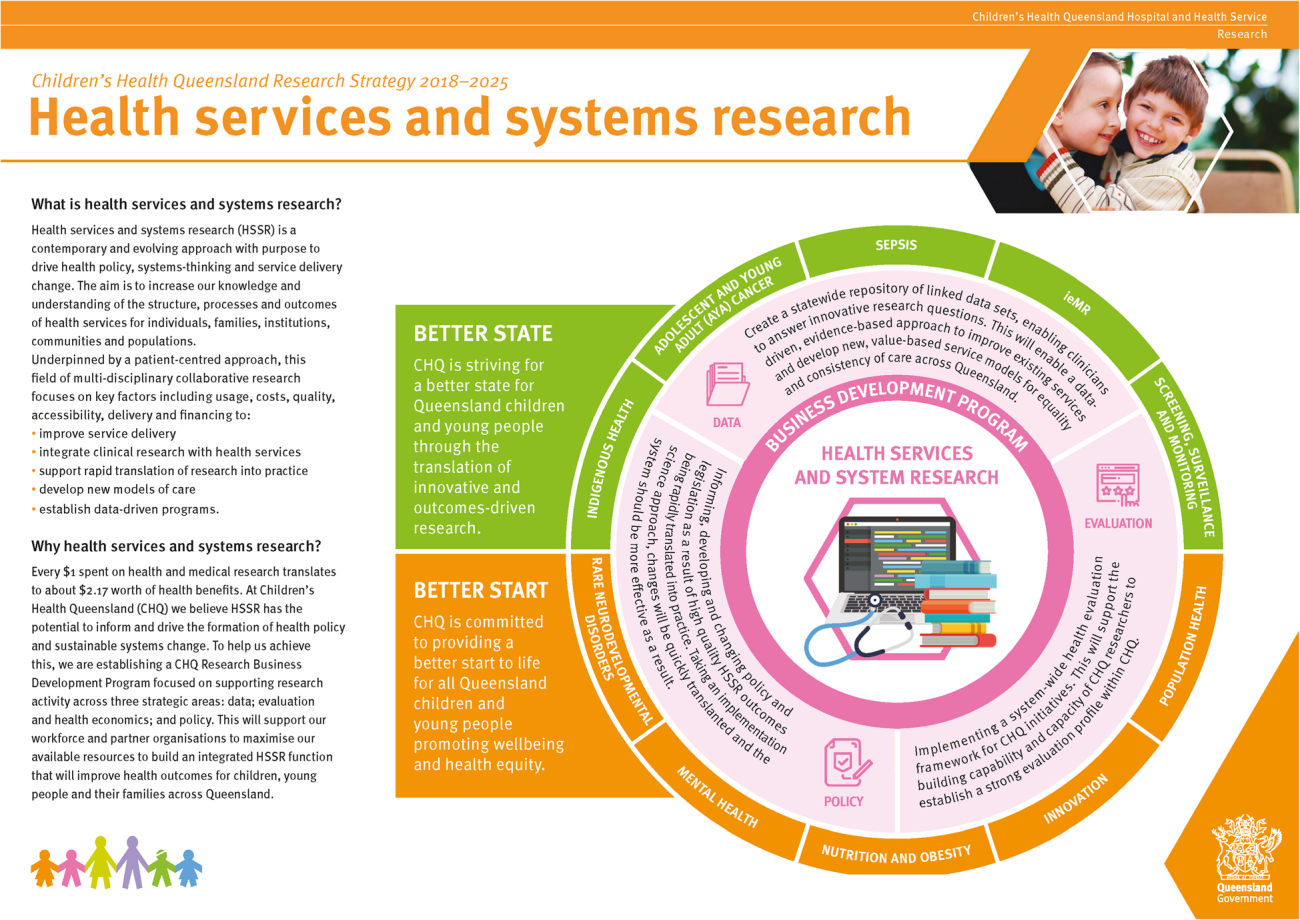
Children’s Health Service and System Research Strategy (CHSSR-S) including Research Priorities and Research Enablers. Source: Children’s Health Queensland Hospital and Health Service. Research Strategy 2018–2025. Department of Health, Queensland Government. 2018

The CHSSR-S (see Fig. 2 for a visual framework, including Research Priorities and Research Enablers) is a novel paediatric-specific, health system-targeted HSSR strategy, co-designed and integrated within an Australian statewide hospital and health service following a participatory systems approach, underpinned by robust consultation with Executive, multidisciplinary, clinical, system and academic research leaders and stakeholders.

Overall, ten Research Priorities and three Research Enablers comprised the CHSSR-S. See Table 2 for a description of all final CHSSR-S Research Priorities, Research Enablers and their alignment with the dimensions of HSSR care [10].

**Table 2:** CHSSR-S Research Priorities and Research Enablers according to HSSR dimensions of care^10^

Domain^10^	Research Priorities and Enablers	Purpose^a^
	Research Priorities	
*Decision-making*	Population Health	Implementing an integrated health service and consumer education improvement agenda to tackle long-term health and disease risk factors.
*Decision-making*	Screening, surveillance, and monitoring	Implement improved system-wide screening programs for disease to deliver the right prevention and early intervention care at the right time, and integrate statewide surveillance and monitoring programs to improve continuity of care
*Decision-making*	Innovation	Integrate a commitment to health service and product innovation that is underpinned by interdisciplinary research and rapid translation into practice, with high quality projects involving newborn screening data, hearing data, medical screening and EMR data sets.
*Implementation*	AYA Cancer	Create a multidisciplinary and integrated system of care and services across the organisation via a newly-developed governance structure and extensive consumer engagement.
	Indigenous Health	Improve access to specialist care in underserved communities for priority Indigenous populations, driven by community engagement and co-design
	Mental Health	Develop a robust service evaluation framework and improvement strategy for mental health services within the health system, driven by academic collaboration.
	Nutrition and Obesity	Drive three statewide HSSR-specific programs to improve nutrition and obesity-related outcomes: Prevention; Treatment; and Education and Training.
	Rare Neurodevelopmental Disorders	Build collaborations with intra-organisational departments to commence new, health service-focused and outcomes-driven clinical trials to inform future service provision for children with rare neurodevelopmental disorders.
	Sepsis	Implement process and outcome evaluation for a multi-system, paediatric sepsis Quality Improvement Project to identify health service gaps and maximise effective, sustainable sepsis treatment programs on a state level.
*Evaluation*	EMR	Developing a point of access to “big data” within a health system EMR to create and investigate innovative research questions and conduct data-driven trials.
	**Research Enablers**	
*Decision-making*	Data	Create an organisation-wide repository of linked data sets, enabling clinician-researchers to develop and answer innovative research questions and implement new, value-based service models
*Implementation*	Policy	Informing, developing and changing policy and legislation secondary to rapidly translated HSSR.
*Evaluation*	Evaluation and Health Economics	Implementing a system-wide health evaluation framework to support capability and capacity building of researchers

*CHSSR-S* Child Health Services and Systems Research Strategy, *AYA* Adolescent and Young Adult, *HSSR* Health Services and Systems, *EMR* electronic medical record

^a^Specific to CHQHHS

Three Research Priorities (Population Health, Screening, Surveillance and Monitoring, Innovation) and one Research Enabler (Data) aligned with the *decision-making* dimension of HSSR care [10]. Six Research Priorities (AYA Cancer, Indigenous Health, Mental Health, Nutrition and Obesity, Rare Neurodevelopmental Disorders, Sepsis) and one Research Enabler (Policy) aligned with the *implementation* dimension of HSSR care [10]. One Research Priority (EMR) and one Research Enabler (Evaluation and Health Economics) aligned with the *evaluation* dimension of HSSR care [10].

Key changes from Research Priorities and Enablers identified in Phase 1 consultation included: (a) Merging - “Nutrition” & “Obesity” Research Priorities; (b) Adding - Research Priority – Screening, Surveillance and Monitoring; (c) Adding - Research Priority – Innovation; (d) Adding - Research Enabler – Evaluation & Health Economics; (e) Merging - Interprofessional Education under the auspice of Research Priority “Innovation”. Overall, these changes were based on:

- The strength of research programs and their clinical and research leaders (a)
- Alignment with the hospital and health service strategic plan (c, e)
- Ability to underpin, support and advance HSSR across multiple Research Programs (b, c, d)

Final membership of the CHSSR-S Steering Committee is presented in Table 3, along with each members’ alignments to Research Priorities, Research Enablers or operational roles. The main additions to the CHSSR-S Steering Committee compared to the HSSR Working Group included operational leaders (e.g. Business Manager, Health Organisation Strategy and Planning, and HSSR Project Officer) and independent, overarching Nursing, Allied Health and Medical Leads. Operational leaders were appointed to support the implementation of the CHSSR-S in partnership with members of the CHSSR-S Steering Committee.

**Table 3:** Final membership of the CHSSR-S Steering Committee and CHSSR-S changes from Phase 1

Leaders and stakeholders	*n* (%)	Research Priority Lead	Research Enabler Lead	Changed from Phase 1?
HSSR Champion	1 (6)	–	Policy	
Executive Leader	2 (12)		–	
Allied Health	1	Innovation	–	✓
Organisational Strategy and Planning	1	*Operational*	–	✓
Research Leader^a^	2 (12)	Mental Health AYA Cancer	–	
Clinical Lead - Allied Health^b^	2 (12)	Nutrition & Obesity Population Health	–	✓
Clinical Lead - Nursing	1 (6)	Screening, Surveillance, Monitoring	–	✓
Clinical Lead - Medical	2 (12)	Sepsis	Evaluation and Health Economics	✓
Data analyst	1 (6)	EMR	Data	
Clinical Research Coordinator	1 (6)	Rare Neurodevelopmental Disorders	–	
Multicultural Health Coordinator	1 (6)	Indigenous Health	–	
Secretariat (Research Support)	1 (6)	*Operational*	–	✓
Media & Communications Officer	1 (6)	*Operational*	–	✓
Project Officer - HSSR	1 (6)	*Operational*	–	✓
Business Manager - Research	1 (6)	*Operational*	–	✓

*CHSSR-S* Child Health Service and System Research Strategy, *HSSR* Health Services and Systems Research, *AYA* Adolescent and Young Adult, *EMR* Electronic Medical Record

^a^Professor (Mental Health) and Research Fellow (AYA Cancer)

^b^Dietitian and Speech Pathologist

### CHSSR-S health system integration

The CHSSR-S was integrated in the health system through the following actions:

1. Embedment within the overall CHQ organisational Research Strategy and Strategic Plan as an independent theme
2. Adapting and implementing an existing health services research impact framework [19] to create a CHSSR-S Actions and Reporting Template (see Table 4), increasing accountability for Research Priority and Research Enabler leads. Research impact measures were based on four broad areas of impact: (a) Research; (b) System; (c) Policy; and (d) Societal, with associated KPI evidence consisting of: research activities; collaboration depth and potential; funding applications; funding awarded; research outputs, including journal article and book publications, conference contributions, theses, and media-related engagement; research group or individual awards; and studentship, including doctoral, Masters and Honours-level students.
3. Integrating responsibility for CHSSR-S implementation within relevant health system employee role descriptions;
4. Allocation of role titles (i.e. “CHSSR-S Research Priority Lead – Population Health”) related to the CHSSR-S for relevant members of the CHSSR-S Steering Committee to promote ownership, responsibility and awareness of CHSSR-S implementation.

**Table 4:** Actions and Reporting Template for each Research Priority and Research Enabler for the integration of the CHSSR-S **(**adapted from Buykx et al.^19^)

Area of impact	Outcome
Research	• Key research questions
	• Key objectives (short/medium/long term)
	• Internal and external collaborations or partnerships
	• Grant application status and future targets
	• Funding awarded
	• Studentship (PhD, Masters)
	• Dissemination plan
	Presentations - conferences, symposiums
	Publications
	Awards
System	• Statement of overall translational impact to the health system
	• Statement of local impact
	• Statement of value-add to the health system
	• Governance structure
	• Metrics of current projects e.g. population reach, clinical and consumer engagement
Policy	• Active participation in policy networks (e.g. advocacy, advisory groups)
	• Key objectives for short/medium/long term policy changes
Societal	• Media engagement and output
	• Evidence of population effect e.g. patient stories, focus groups, media releases

*CHSSR-S* Child Health Services and Systems Research Strategy

## Discussion

### Main findings and significance

The CHSSR-S is a children’s health service and system-integrated HSSR strategy with the specific purpose of creating impactful health service improvements across ten Research Priorities, supported by three Research Enablers, that are translated rapidly into improved patient outcomes on a population level.

The novelty of the CHSSR-S is its intrinsic nature within a statewide hospital and health service, rather than attempting to drive change from a position that is external to the system. Intra-organisational integration of the CHSSR-S supports rapid, evidence-based health system and policy change, and can be quantified by using an appropriate evaluation framework [19]. It creates a conduit to map system HSSR capability and capacity, identify research areas of strength and weakness, rapidly translate research into practice, encourage robust HSSR and directly influence health policy from within the system.

In the CHSSR-S, Research Priority leaders are multidisciplinary and interdisciplinary clinician-researchers and are well-positioned to recognise health service gaps and support outcomes-driven clinical research translation. High quality, contextualised and consumer-led HSSR research can be quickly translated into clinical system change, with the real-time demonstration of improved health outcomes via the unique partnership of on-the-ground clinician-researchers and the system.

### Comparison with previous HSSR agendas

Many studies relating to HSR describe its application to a specific medical condition rather than the broader health system [21–23]. developed Previous paediatric HSR agendas have primarily been conceptual models, have demonstrated limited engagement or integration in the health “system” and are specific to the United States [12, 24, 25]. Broad actions are typically emphasised as priorities, such as increasing the size and capacity of the children’s HSR workforce, and evaluating the quality and safety of health care delivery [24, 25]. These models are also now outdated. Health services research has only recently emerged as a priority for global health systems [15] and secondary to statistical, technological and infrastructure advancements, requires consideration of advancements in applied health economics, biostatistics, “big data”, and electronic medical record and patient management systems, as examples.

A promising and recent (2017) example of a paediatric HSR agenda is that developed in the USA by Fairbrother et al. [12] Engagement with leaders in child health research, clinicians, system leaders, policy makers and consumers resulted in the development of six high-priority research domains for paediatric health services research. The research domains present a broad framework that can be adapted and used by funding bodies and researchers to positively influence paediatric health services and policy. Despite this, their strategy was not purposed for integration within a paediatric health system – the translational setting where the true impact of a HSSR strategy can be observed. The advantage of the CHSSR-S is that it provides a strategy that has been integrated into an existing health system to guide other health services globally on developing and translating local HSSR strategies to improve health service delivery, quality, safety and health outcomes.

### Core principals of modern HSSR

It is well understood that Australian health services, in their current fragmented form, exhibit significant structural and systemic challenges that remain unsustainable for delivering safe, quality and efficient healthcare [26]. Conducting HSSR is critical to current health organisations throughout every level of the system (micro, macro, and meso) to understand the true cost and impact of services being delivered for health system optimisation [15].

Technical advances in patient management systems, EMRs and informatics generates opportunities to create novel research questions that address persisting and novel HSSR problems. The strongest motivator for implementing a health system-integrated HSSR Strategy is to ensure health service demand is met and wastage within the system is minimised; approximately 30% of all health expenditure is currently wasted within the system [27]. There is global urgency for a shift towards value-based care within the health system [8, 9] and system-integrated, action- and value-based HSSR is required to maximise sustainable, high-quality healthcare and the likelihood of meeting future service demand.

### Implications for future research

Robust evaluation of any health system-wide HSSR strategy is essential to identifying enablers of success, implementation gaps, and opportunities for further service optimisation and research. Determining the implementation success of a HSSR strategy would likely be strengthened if assessed through a framework for implementation outcomes in conjunction with HSSR-relevant KPIs (such as those presented in Table 4). Such a framework is offered by Proctor et al. [28] through eight implementation outcomes, including: acceptability, adoption, appropriateness, feasibility, fidelity, implementation cost, penetration and sustainability.

There is also strong advocation for implementation science theory to guide and evaluate practice changes [29]. Using a framework for evaluating implementation outcomes, in combination with appropriate implementation science theory relevant to health systems, such as the Normalisation Process Theory [29, 30] or Organisational Readiness to Change [31], would provide a robust approach to evaluating impact and sustainability of health system-integrated HSSR strategy such as the CHSSR-S. Enablers and barriers of impactfully translating a HSSR strategy into routine practice could then be identified and addressed to encourage further uptake and normalisation by clinicians and researchers.

### Strengths and weaknesses

Strengths of CHSSR-S development included: (1) An iterative design approach; (2) Endorsement by an organisation-wide steering committee with internal and external research leader and stakeholder representation; and (3) A dedicated, executive-level HSSR “Champion” who delivered system-wide leadership. Strengths of CHSSR-S health system integration included: (1) Adaptation of a HSSR research impact evaluation framework to quantify impact of the CHSSR-S [19]; and (2) Embedment within organisational-level strategic plans to maximise compliance and the opportunity for sustainability.

During the initial phases of the CHSSR-S development, clinician understanding of HSSR was an identified gap. Clinicians struggled to understand its definition, purpose, depth of applicability and potential to influence healthcare on a system-level. The relative novelty of HSSR within the health system [15] and a lack of clinician-researcher engagement was a potential explanation for this. Successful implementation of a health service innovation requires end-user awareness and understanding – therefore, a system-integrated HSSR strategy requires healthcare worker education to maximise likelihood of its adoption [20]. Whilst increasing the HSSR knowledge-based capacity of on-the-ground clinicians and healthcare workers is an essential first step, translating this knowledge into action-based capacity is a long-term goal and is indicative of true system integration.

## Conclusions

We have developed and integrated the first known Children’s Health Service and System Research Strategy (CHSSR-S) within a dedicated paediatric health system (CHQ). The CHSSR-S demonstrates real-world integration and translation into the healthcare sector to improve child health across a broad range of Research Priorities, enabled by modern Research Enablers. Global health systems may adapt the process described in this study to develop and integrate local and contextualised HSSR strategies to support their patients, clinicians, researchers and services. Robust process, outcome and economic evaluation of the implementation of the CHSSR-S across CHQ is now critical to generate a foundational evidence base for the success of a health system-integrated HSSR strategy.

Health systems need to adapt to increasing health service demands. Modern HSSR strategies require health service integration, an interprofessional and multidisciplinary approach, improved knowledge- and action-based capacity of on-the-ground health professionals, and dedicated HSSR and Executive-level “Champions” to leverage internal system change. Any innovative change that arises secondary to HSSR must retain key principles, including:

1. The right of every child and family to safe and quality care;
2. Data-informed and evidence-based healthcare delivery; and
3. Service provision that is accessible, equitable, and contextualised across all populations, especially underserved and priority populations, to maximise the equity of healthcare delivery.

We recommend that global hospitals and health services invest in first understanding HSSR in their current system, with the intention of building, integrating and implementing a strategic, system-wide HSSR direction that sustainably services the pursuit of excellence in healthcare quality, safety and equity.

## Supplementary information

**Additional file 1.**

## Data Availability

Data sharing is not applicable to this article as no datasets were generated or analysed during the current study.

## Abbreviations

CHSSR-S: Children’s Health Service and System Research Strategy
HSR: Health Services Research
MRFF: Medical Research Futures Fund
HSSR: Health Services and Systems Research
KPI: Key Performance Indicator
AYA: Adolescent and Young Adult
EMR: Electronic Medical Record
