# Correction to: Building a Children’s Health Service and System Research Strategy: development and integration in an Australian paediatric healthcare setting

**DOI:** 10.1186/s12913-020-05509-7

**Published:** 2020-07-08

**Authors:** Robyn Littlewood, Oliver J. Canfell, Frank Tracey

**Affiliations:** 1 Health and Wellbeing Queensland, Queensland Government, The State of Queensland, 139 Coronation Drive, Milton, QLD 4064 Australia; 2 School of Human Movement and Nutrition Sciences, Faculty of Health and Behavioural Sciences, The University of Queensland, St Lucia, QLD 4067 Australia; 3 Children’s Health Queensland Hospital and Health Service, Department of Health, Queensland Government, The State of Queensland, South Brisbane, QLD 4101 Australia; 4 Faculty of Medicine, The University of Queensland, St Lucia, QLD 4067 Australia

**Correction to: BMC Health Serv Res 20, 589 (2020)**

**https://doi.org/10.1186/s12913-020-05267-6**

Following publication of the original article [1], an error was identified in the Design section under Methods. A sentence “(3) Integration of the HSSR strategy within the health system” is placed in the wrong place. It should be placed next to “(2) Development of the CHSSR-S strategy” instead of “3. Evaluation – what are the outcomes resulting from this care?”.

The updated paragraphs are given below and the changes have been highlighted in **bold typeface**.

Design

Development of the Children’s Health Service and System Research Strategy (CHSSR-S) was informed by an inductive, bottom-up, participatory systems approach. This conceptual approach intended to place clinical, research and system leadership at the forefront of designing and implementing the CHSSR-S [16, 17]. This study was conducted across three key phases that were underpinned by our conceptual approach (see Fig. 1 for an overview of the purpose, participants and methodology of each phase):

1. *Identifying the current state of HSSR within the health system*
2. *Development of the CHSSR-S strategy*
3. ***Integration of the HSSR strategy within the health system***

The overall structure and content of the CHSSR-S was aligned with three interrelated dimensions of care relevant to HSSR [10]:

1. Decision-making – what is the best care to provide?
2. Implementation – how can we best provide this care?
3. Evaluation – what are the outcomes resulting from this care?

The original article [1] has been corrected.
