# Supplementary material for: Building a Children’s Health Service and System Research Strategy: development and integration in an Australian paediatric healthcare setting
Source: BMC Health Serv Res. 2020 Jun 29;20:589. doi: 10.1186/s12913-020-05267-6 (PMC7322850; doi:10.1186/s12913-020-05267-6)
Supplement: Supplementary file 1 — Additional file 1. [file 12913_2020_5267_MOESM1_ESM.docx]

## Research Leaders and Stakeholders – Questions to drive development of the Children’s Health Service and Systems Research Strategy (CHSSR-S)

Robyn Littlewood, Oliver J Canfell, Frank Tracey

- How can you change the system?
- What outcomes would you like to improve?
- What service gaps can you identify?
- How can you improve the value of your service?
- How can you influence statewide and national policy?
- How can you use innovation and digital health to evaluate services and inform delivery of care?
- Who can drive robust evaluation of your current services?
